# Four Novel Variants in *POU4F3* Cause Autosomal Dominant Nonsyndromic Hearing Loss

**DOI:** 10.1155/2020/6137083

**Published:** 2020-07-01

**Authors:** Tian-Yi Cui, Xue Gao, Sha-Sha Huang, Yan-Yan Sun, Si-Qi Zhang, Xin-Xia Jiang, Yan-Zhong Yang, Dong-Yang Kang, Qing-Wen Zhu, Yong-Yi Yuan

**Affiliations:** ^1^College of Otolaryngology Head and Neck Surgery, Chinese PLA General Hospital, Chinese PLA Medical School, 28 Fuxing Road, Beijing 100853, China; ^2^National Clinical Research Center for Otolaryngologic Diseases, State Key Lab of Hearing Science, Ministry of Education, China; ^3^Beijing Key Lab of Hearing Impairment Prevention and Treatment, Beijing, China; ^4^School of Basic Medical Sciences, Henan University, Kaifeng 475001, China; ^5^Department of Otolaryngology, PLA Rocket Force Characteristic Medical Center, 16# XinWai Da Jie, Beijing 100088, China; ^6^Department of Otolaryngology Head & Neck Surgery, The Second Hospital of Hebei Medical University, Heping West Road No. 215, Shijiazhuang, Hebei 050000, China

## Abstract

Hereditary hearing loss is one of the most common sensory disabilities worldwide. Mutation of POU domain class 4 transcription factor 3 *(POU4F3*) is considered the pathogenic cause of autosomal dominant nonsyndromic hearing loss (ADNSHL), designated as autosomal dominant nonsyndromic deafness 15. In this study, four novel variants in *POU4F3*, c.696G>T (p.Glu232Asp), c.325C>T (p.His109Tyr), c.635T>C (p.Leu212Pro), and c.183delG (p.Ala62Argfs∗22), were identified in four different Chinese families with ADNSHL by targeted next-generation sequencing and Sanger sequencing. Based on the American College of Medical Genetics and Genomics guidelines, c.183delG (p.Ala62Argfs∗22) is classified as a pathogenic variant, c.696G>T (p.Glu232Asp) and c.635T>C (p.Leu212Pro) are classified as likely pathogenic variants, and c.325C>T (p.His109Tyr) is classified as a variant of uncertain significance. Based on previous reports and the results of this study, we speculated that *POU4F3* pathogenic variants are significant contributors to ADNSHL in the East Asian population. Therefore, screening of *POU4F3* should be a routine examination for the diagnosis of hereditary hearing loss.

## 1. Introduction

Hearing loss is one of the most common hereditary sensory disabilities worldwide [1]. Hair cells (HCs) in the inner ear are critical for hearing ability. HCs transfer the mechanical vibration into an acoustic electrical signal, which can then be transmitted to the auditory cortex via spiral ganglion neurons (SGNs) [2]. The causes of deafness are complex, and most of the hearing loss is due to irreversible HCs loss. HCs are very sensitive and vulnerable to many stresses and damage, which can be divided mainly into genetic factors, environmental factors, ototoxic drugs, aging, inflammation, and other unknown etiologies [3–5]. Among all these factors, it is estimated that genetic factors account for more than 50% of the causes of deafness [6]. Hereditary hearing loss can be classified as syndromic hearing loss or nonsyndromic hearing loss (NSHL) according to whether the patient has other symptoms or signs, and these account for 30% and 70% of cases of hearing loss, respectively [7]. NSHL can be further divided into three categories according to the mode of inheritance: autosomal dominant nonsyndromic hearing loss (ADNSHL), autosomal recessive nonsyndromic hearing loss, and X-linked nonsyndromic hearing loss. ADNSHL accounts for 15% of cases of NSHL [8]. One of the most significant characteristics of hereditary hearing loss is a high degree of heterogeneity. To date, 49 genes related to ADNSHL, including POU domain class 4 transcription factor 3 (*POU4F3*) and approximately 70 other loci, have been reported (http://hereditaryhearingloss.org/).

The *POU4F3* gene encodes POU4F3, a POU-domain class IV protein, has two exons, and encodes a protein of 338 amino acids that belongs to the POU-domain family of transcription factors, which are expressed specifically in inner ear hair cells and play a critical role in the maturation, differentiation, and maintenance of inner ear hair cells [9, 10]. POU4F3 contains two conserved DNA-binding domains (a POU-specific domain and a POU homeodomain), which are the main functional parts [10].

In 1998, *POU4F3* was first described as a disease-causing gene within the DFNA15 locus in an Israeli Jewish family [11]. To date, 32 variants (including those in this study) and whole-gene deletion of *POU4F3* have been reported to cause ADNSHL with variable ages of onset and degrees of severity in various ethnic groups, including Chinese, Japanese, Dutch, Korean, and Brazilian populations [12–24]. In 2017, Kitano et al. reported that *POU4F3* variants represent the third largest cause of ADNSHL (2.5%, 15/602) in Japan and the most prevalent configuration as midfrequency hearing loss type followed by high-frequency hearing loss [14]. He et al. reported that the *POU4F3* pathogenic variant is a relatively common (3/18) cause of ADNSHL among Chinese Hans [15]. Therefore, impairment of hair cells in the cochlea caused by pathogenic variants of *POU4F3* has been considered as one of the major causes of sensorineural hearing loss [14].

In this study, we identified four novel variants using targeted next-generation sequencing (NGS) of a panel of 168 deafness genes from four different Chinese families suffering from ADNSHL. Among the four novel variants, three are missense variants, c.696G>T (p.Glu232Asp) detected in family A, c.325C>T (p.His109Tyr) in family B, and c.635T>C (p.Leu212Pro) in family C, and the fourth is a frameshift variant, c.183delG (p.Ala62Argfs∗22), which was identified in family D. Hearing loss in the four families analyzed in this study showed a high degree of variability, even in patients carrying the same variant within one family.

## 2. Materials and Methods

### 2.1. Subjects

**Figure 1:**
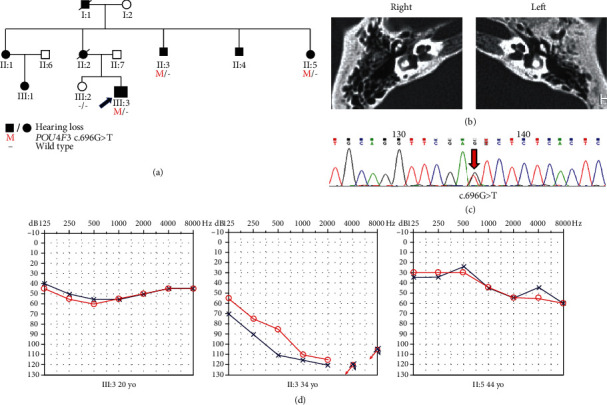
Pedigree, temporal bone CT, variant analysis, and audiogram of family A. (a) Affected subjects are denoted in black. Arrow shows the proband. (b) Temporal bone CT of the III:3 shows no structural change. (c) Chromatogram shows *POU4F3* heterozygous c.696G>T detected in patients. (d) Audiograms of the affected subjects. Hearing loss appears to be highly heterogeneous (red: right ear; blue: left ear).

**Figure 2:**
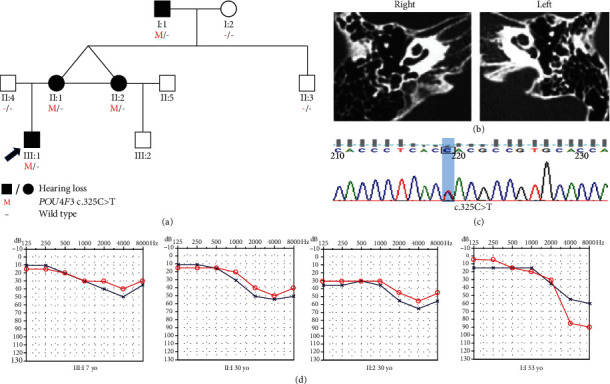
Pedigree, temporal bone CT, variant analysis, and audiogram of family B. (a) Affected subjects are denoted in black. Arrow shows the proband. (b) Temporal bone CT of the III:1 shows no structural change. (c) Chromatogram shows *POU4F3* heterozygous c.325C>T detected in patients. (d) Audiograms of the affected subjects. Hearing loss appears to involve high frequency (red: right ear; blue: left ear).

**Figure 3:**
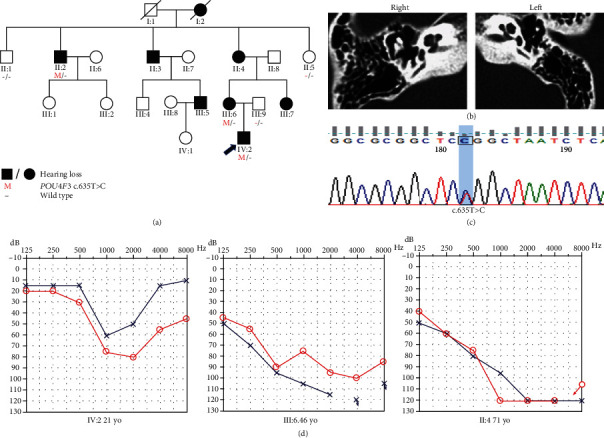
Pedigree, temporal bone CT, variant analysis, and audiogram of family C. (a) Affected subjects are denoted in black. Arrow shows the proband. (b) Temporal bone CT of the IV:2 shows no structural change. (c) Chromatogram shows *POU4F3* heterozygous c.635T>C detected in patients. (d) Audiograms of the affected subjects. Audiogram configuration of IV:2 was U-shaped. Downsloping audiogram configurations were observed in III:6 and II:4 (red: right ear; blue: left ear).

**Figure 4:**
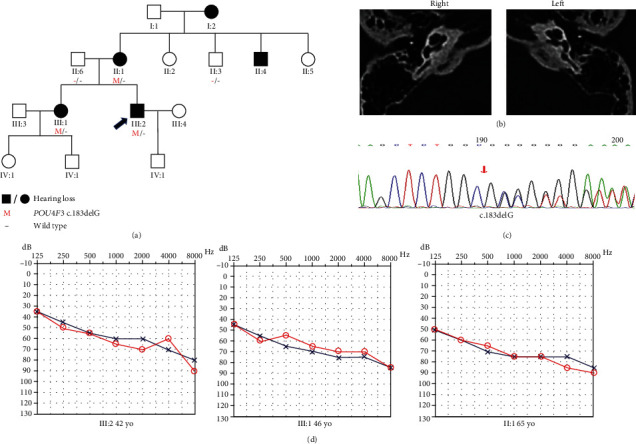
Pedigree, temporal bone CT, variant analysis, and audiogram of family D. (a) Affected subjects are denoted in black. Arrow shows the proband. (b) Temporal bone CT of the III:2 shows no structural change. (c) Chromatogram shows *POU4F3* heterozygous c.183delG detected in patients. (d) Audiograms of the affected subjects (red: right ear; blue: left ear).

Probands suffering from ADNSHL in the four families were recruited from the Chinese PLA General Hospital. The pedigrees of these four families are shown in Figures 1–4(a) In addition to the probands, three additional members of family A (II:3, II:5, and III:2), six additional members of family B (I:1, I:2, II:1, II:2, II:3, and II:4), five additional members of family C (II:1, II:2, II:5, III:6, and III:9), and four additional members of family D (II:1, II:3, II:6, and III:1) were recruited from our hospital. All of the subjects or their guardians provided written informed consent to participate in the study. This study was approved by the Ethics Research Committee of the Chinese PLA General Hospital.

### 2.2. Clinical Information and Examination

Clinical information was obtained via multiple interviews with the subjects. Medical history was obtained using a questionnaire that elicited responses regarding the symmetry of hearing loss, subjective degree of hearing loss, use of hearing aids, age at onset, evolution, presence of tinnitus, noise exposure, medications, trauma history, and other relevant clinical manifestations. The subjects all received clinical examinations at the Department of Otorhinolaryngology, which included otoscopy, physical examination, pure tone audiometric examination (at frequencies from 125 to 8000 Hz), computed tomography scans of the temporal bone, and acoustic immittance testing. The tandem gait test was performed to evaluate the balance. The diagnosis of sensorineural hearing loss was made according to the WHO criteria based on audiometric examination performed as described previously (the methods description partly reproduces our wording) [12]. Tandem gait and Romberg tests were performed to evaluate balance.

### 2.3. Variant Analysis

DNA was extracted from peripheral blood samples from all subjects using a blood DNA extraction kit (TIANGEN, Beijing, China), according to the manufacturer's instructions.

The most prevalent genes related to hearing loss, including *GJB2*, *SLC26A4*, and *mtDNA12SrRNA*, were screened in all of the probands and Chinese controls. The probands and some of the additional family members were examined using a gene panel containing 168 genes related to deafness (Supplementary Table 1). Capture sequencing and NGS of the coding exons of the 168 deafness-related genes and their flanking 100 bp were performed on the Illumina HiSeq 2000 (Illumina, San Diego, CA, USA) using the MyGenostics gene enrichment system (MyGenostics, Boston, MA, USA).

The methods for DNA library preparation, amplification, capture, detection, sequencing, and bioinformatics analyses were described previously [12]. Nonsynonymous variants were further evaluated for candidate pathogenic variants. Variants were annotated by ANNOVAR; compared with multiple databases including gnomAD, dbSNP, and ExAC; and were predicted by the computational programs SIFT, PolyPhen-2, and MutationTaster. Potential pathogenic variants were filtered using a minimum allele frequency threshold ≤ 0.001 for dominant inheritance [25]. As *POU4F3* has an autosomal dominant inheritance pattern, only heterozygous subjects were selected.

Manual classification of those variants was conducted based on American College of Medical Genetics and Genomics (ACMG)/Association for Molecular Pathology (AMP) guidelines for genetic hearing loss [26]. Sanger sequencing was performed in members of the four families, and the candidate variant of each family was cosegregated with the hearing loss phenotype.

## 3. Results

### 3.1. Families and Clinical Characteristics

The pedigrees of the four families showed autosomal dominant inheritance patterns (Figures 1–4(a)). High-resolution CTs of the temporal bone in probands of four families were normal, excluding middle- and inner-ear malformations (Figures 1–4(b)). The hearing impairments in these four families were sensorineural, postlingual, late onset, and progressive. Audiograms of some affected members of these four families are shown in Figures 1–4(d).

Family A was a three-generation Chinese family with ADNSHL and included eight affected patients (Figure 1). The ages at onset of the subjects ranged from 7 to 22 years old. The audiogram of the 20-year-old proband (III:3) with an onset age of 13 years showed all-frequency moderate hearing loss. The audiogram of II:3 showed profound hearing loss; interestingly, this subject had an onset age of 7 years old, which was the earliest in this family. The audiogram of II:5 showed a moderate level of hearing loss.

Family B was a three-generation Chinese family with ADNSHL and included four affected patients (Figure 2). The audiograms had a downsloping shape. The hearing loss in family B involved mostly high frequencies. The proband (III:1) was 7 years old with symmetric hearing loss, and the audiogram showed mild hearing impairment; thus, the proband could communicate with others normally. This family included one set of affected identical twin sisters (II:1 and II:2) who had similar audiograms but different hearing thresholds. Comparison of the audiograms of the proband and 53-year-old I:1 showed that although the hearing impairment had progressed over time, the progression was slight in the affected individual I:1 and involved mainly high frequencies.

Family C was a four-generation Chinese family with ADNSHL and included eight affected patients (Figure 3). The audiogram of proband (IV:2) was asymmetric, and hearing loss involved mainly middle frequencies. Hearing impairment in family C was postlingual, with onset in the first or second decade of life and progression to profound deafness with advancing age. The onset age of the proband was 15 years, and hearing loss was progressive. There was no history of hearing aid use or artificial cochlear implants in the proband. With regard to other auditory symptoms, the proband had complained of tinnitus. Audiograms showed that although low-frequency and high-frequency hearing were normal in the beginning, hearing ultimately deteriorated at all frequencies in the order of middle, high, and low frequencies. Downsloping audiogram configurations were observed in two subjects, who were 46 (III:6) and 69 (II:4) years old, whereas the audiogram of IV:2 was U-shaped (Figure 3(d)). Audiograms were unavailable for the other affected subjects.

Family D was a four-generation Chinese family with ADNSHL and included five affected patients (Figure 4). The audiogram of the proband (III:2) had a downsloping shape. The hearing impairment of the proband was moderate. The proband had a history of using hearing aids, but the effect was unsatisfactory. With regard to other auditory-related symptoms, individual II:1 and the proband complained of tinnitus.

### 3.2. Variant Identification

**Figure 5:**
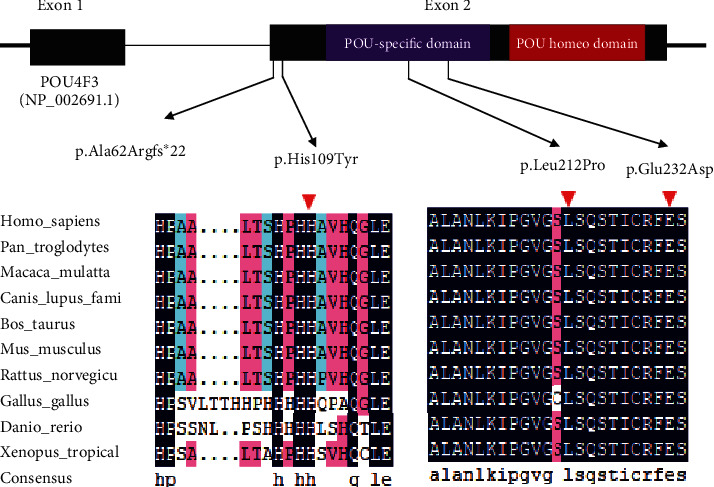
Protein structure of POU4F3 and conservation analysis. (a) Domain structure of POU4F3 showing the localization of four variants identified in this study. (b) Protein alignment showing that POU4F3 p.His109Tyr, p.Leu212Pro, and p.Glu232Asp all occur at evolutionarily conserved amino acids (shown by the red triangle) across 10 species.

According to the autosomal dominant pattern of inheritance, only variants that were heterozygous in the affected siblings were selected as candidates. Four novel variants were identified using targeted NGS of 168 known deafness-related genes in the four different ADNSHL Chinese families. Among the four novel variants, three were missense variants: c.696G>T (p.Glu232Asp) detected in family A, c.325C>T (p.His109Tyr) in family B, and c.635T>C (p.Leu212Pro) in family C. The fourth variant was a frameshift variant, c.183delG (p.Ala62Argfs∗22), which was identified in family D. Sanger sequencing was performed in the other participating family members from these four families, which confirmed that these variants cosegregated with the hearing phenotypes (Figures 1–4(c)). The four variants have not been reported in previous studies and were not detected in 481 Chinese controls with normal hearing. The variants c.696G>T (p.Glu232Asp), c.635T>C (p.Leu212Pro), and c.183delG (p.Ala62Argfs∗22) are not present in the gnomAD or ExAC database, and c.325C>T (p.His109Tyr) has an allele frequency of 0.0001 in both gnomAD (Asian) and ExAC (Asian). The localizations of the four novel variants are shown in Figure 5(a). Conservation analysis was performed in the three families with missense variants (Figure 5(b)) and showed that the three variants are conserved among 11 species. Finally, the four novel variants were predicted to be deleterious by SIFT, Polyphen2, and CADD software. According to the American College of Medical Genetics and Genomics/Association for Molecular Pathology guidelines for genetic hearing loss [26, 27], c.696G>T (p.Glu232Asp) is classified as a likely pathogenic variant (PM1+PM2+PM5+PP1+PP3), c.325C>T (p.His109Tyr) is classified as a variant of uncertain significance (PP1), c.635T>C (p.Leu212Pro) is classified as a likely pathogenic variant (PM1+PM2+PP1+PP3), whereas c.183delG (p. Ala62Argfs∗22) is classified as a pathogenic variant (PVS1+PM2+ PP1) (Table 1).

## 4. Discussion

In mammals' cochlea, HCs are the key cell type for hearing function, which convert the mechanical vibrations into electronic neural signals [9]. HCs are sensitive to multiple stresses and injuries and are easy to damage. While a mammal's cochlea only has very limited HC regeneration ability, most of the HC damage is permanent and irreversible [28–34]. Genetic factor accounts for 50% of sensorineural hearing loss. A genetic diagnosis is valuable for providing essential prognostic information needed for deciding optimal treatment/rehabilitation options and for genetic counseling [35]. Molecular epidemiological studies have found several common deafness genes in Chinese deafness population, such as *GJB2*, *SLC26A4*, and *mtDNA12SrRNA* [36]. However, genetic variants responsible for a large number of cases of hereditary hearing loss remain unknown. Next-generation sequencing has greatly increased the efficiency in screening known deafness genes for diagnostic purposes and in identifying new deafness genes [37–40].

In this study, we identified four novel variants in the *POU4F3* gene, three missense variants, and one frameshift variant, which led to sensorineural hearing loss in four different Chinese families. The variabilities in onset age and severity of hearing loss in these four families demonstrated the heterogeneity of these variants both interfamilial and intrafamilial.

In 1998, *POU4F3* was first discovered in an Israeli Jewish family. The results of a linkage analysis identified it as a novel independent locus for hearing loss, and the gene was designated as autosomal dominant nonsyndromic deafness 15 (DFNA15) [11]. The clinical presentation of DFNA15 is a form of progressive nonsyndromic sensorineural hearing loss with postlingual onset [13, 41]. In the present study, the earliest recorded age of hearing loss onset in affected individuals was 7 years old (III:1, family B). Among the 32 variants, 28 were reported in East Asian populations (13 in Japan, 12 in China, and 3 in Korea), and only 4 variants (2 in Netherlands, 1 in Israel, and 1 in Brazil) were reported from other areas, indicating that the *POU4F3* pathogenic variant is an important contributor to ADNSHL, especially in East Asian populations (Table 2). In summary, the variant of *POU4F3* is relatively common, especially in East Asian populations. Therefore, screening of *POU4F3* should be a routine examination for the diagnosis of hereditary hearing loss. *POU4F3* contains only two exons, making it convenient for screening.

Hearing impairment involves mainly the middle frequency range (1000–2000 Hz) in a low percentage of cases of hereditary hearing loss. Kitano et al. reported that *POU4F3*-associated hearing loss usually presents with middle- or high-frequency hearing loss [14]. In 2018, we reported a family with middle-frequency hearing loss associated with *POU4F3* c.602T>C (p.Leu201Pro) [12]. In this study, the proband in family C presented with typical middle-frequency hearing loss, and the older patients showed downsloping audiograms and mainly middle- and high-frequency hearing loss. In accordance with our previous report, we proposed that the affected frequencies of certain types of *POU4F3*-associated hearing loss were in the order of middle (U-shaped audiogram), high (downsloping audiogram), and low frequencies (flatter audiogram). Accordingly, the different forms of auditory configuration represented different disease phases.

POU4F3 belongs to a family of proteins characterized by a well-conserved bipartite domain [42]. The bipartite domain is comprised of a POU-specific domain (amino acids 179–256) and a POU homeodomain (amino acids 274–333) separated by a linker [43]. These two domains are responsible for the main functions of POU4F3.

However, the specific mechanisms underlying sensorineural hearing loss caused by the *POU4F3* variant have remained unclear to date. Several previous studies have shown that although the wild-type POU4F3 is localized almost exclusively in the nucleus, the mutant protein is also present in both the cytoplasm and the nucleus. Cytoplasmic localization of transcription factors obviously affects their ability to activate downstream targets. Mutant proteins showed greatly reduced capability for binding to DNA as well as transcriptionally activating reporter gene expression [10, 16, 20, 21, 23]. One possible mechanism is that the variant in the POU homeodomain of *POU4F3* leads to a prematurely truncated protein with loss of the second and third helices, and the third helix is crucial for high-affinity binding to DNA; thus, the target gene cannot be induced, leading to impairment of inner ear hair cells [11].

Further studies showed that POU4F3 contains two nuclear localization signals (NLSs): a monopartite NLS (amino acids 274–278) and bipartite NLS (amino acids 314–331) [10]. NLS is crucial for the trafficking of cytoplasmic proteins into the nucleus. Variant of *POU4F3* results in the absence of these two NLSs, which leads to subcellular protein mislocalization. The normal wild-type protein is localized mainly in the nucleus [44]. However, transient transfection studies revealed that NLS-mutated POU4F3 proteins are localized mainly in the cytoplasm, most likely due to the absence of the NLSs. As POU4F3 proteins are transcription factors, their function requires their entry into the nucleus and binding to DNA. In addition, the mutated POU4F3 proteins have longer half-lives and much lower levels of transcriptional activity than those of the wild-type protein [11].

Although mice require only one copy of the functional POU4F3 to retain hearing [45, 46], several previous studies supported that haploinsufficiency is the most likely molecular mechanism underlying the hearing loss caused by the *POU4F3* variant [13, 23, 24]. Heterozygous deletion of the entire *POU4F3* has been reported in a Brazilian family with ADNSHL [13]. Another study identified an ADNSHL-associated *POU4F3* heterozygous frameshift variant c.1007delC (p.Ala336fs∗), which would produce a transcript without an in-frame stop codon, and presumably, the nonstop mRNA might be degraded through nonstop decay [24]. Both variants cause the loss of one copy of POU4F3, indicating the mechanism of haploinsufficiency [47]. Also, the subcellular protein mislocalization of mutant POU4F3 shown in Lin et al. and other studies support the mechanism of haploinsufficiency [10, 16, 20]. ExAC pLI score of POU4F3 is 0.721 which is not an indication for extreme loss of function intolerance. In addition, studies showed that the pathways downstream of POU4F3 play crucial roles in the maintenance of inner ear hair cells, which also provides insight into the mechanisms underlying *POU4F3* mutation-induced hearing loss. A study performed in 2004 showed that the degeneration of outer hair cells caused by the *POU4F3* variant was mainly or entirely the result of inhibited expression of growth factor independence 1 (Gfi1), which is one of the target genes of POU4F3 *[*46*]*. Gfi1 not only plays a late role in the differentiation and maintenance of hair cells but also promotes the formation of hair cells in cooperation with atonal BHLH transcription factor 1 (Atoh1) [48]. In addition, another study showed that Atoh1 is upstream of POU4F3 and Gfi1 [49]. Thus, regulation of Atoh1 will affect the expression of Gfi1, and both Atoh1 and POU4F3 are required for maintenance of Gfi1 expression [50]. Another possible mechanism is that the variant of *POU4F3* inhibits the expression of myosin VI, which plays a large role in the maintenance of stereocilia of hair cells that are responsible for auditory transduction [51]. Tornari et al. reported that the orphan thyroid nuclear receptor Nr2f2, which is related to the development and survival of hair cells, is a target of POU4F3 [52]. Although several downstream pathways and probable mechanisms have been reported, further studies are required to explore the mechanisms related to *POU4F3*.

In this study, we identified four novel variants in *POU4F3* (three missense variants and one frameshift variant) involved in hearing loss. The missense variant c.696G>T (p.Glu232Asp), detected in family A, is located in the POU-specific domain, and a different missense variant at the same locus, c.694G>A (p.Glu232Lys), has been reported previously [22]. The missense variant c.696G>T (p.Glu232Asp) in *POU4F3* leads to substitution of the glutamate at position 232 with an aspartic acid, which probably alters the structure of the *α*-helix of the POU-specific domain. The structural changes in the helix might affect the DNA-binding ability, what was probably responsible for the hearing loss in this family. The missense variant found in family C, c.635C>T (p.Leu212Pro), is also localized in the POU-specific domain, and it is possible that the mechanism of action is likely the same as described above. The missense variant observed in family B, c.325C>T (p.His109Tyr), is located in the transcriptional activation domain, which is not a functional domain, and this is likely why the hearing impairment in this family was mild. This variant is heterozygous in 0.016% (3/18,385 alleles) of East Asians, according to the gnomAD database. We speculate that its detection in the public database is due to the mild hearing loss associated with this variant. The frameshift variant, c.183delG (p.Ala62Argfs∗22), identified in family D results in a truncated protein with loss of both functional domains crucial for high-affinity binding to DNA.

## 5. Conclusions

In summary, four novel variants in *POU4F3* were identified in four different families. These consisted of three missense variants, c.696G>T (p.Glu232Asp), c.325C>T (p.His109Tyr), and c.635C>T (p.Leu212Pro), and one frameshift variant, c.183delG (p.Ala62Argfs∗22). These variants of *POU4F3* are considered to be responsible for ADNSHL, designated as DFNA15. *POU4F3* variants are not rare, and therefore, screening of *POU4F3* should be included in routine examinations for diagnosis of ADNSHL. Further studies are required to determine the specific mechanisms underlying hearing loss.

## Figures and Tables

**Table 1:** Summary of the four *POU4F3* variants identified in this study.

Family	Nucleotide change	Amino acid change	hom/het	Allele frequency^∗^	Pathogenicity	ACMG code	Computational evidence			Origin of variant	Cosegregation
							SIFT	PolyPhen	Mutation assessor		
A	c.696G>T	p.(Glu232Asp)	het	0.0001	Likely pathogenic	PM1+PM2+PM5+PP1+PP3	Deleterious	Probably damaging	High	De novo	Yes
B	c.325C>T	p.(His109Tyr)	het	—	Uncertain significance	PP1	Tolerated	Benign	Medium	De novo	Yes
C	c.635T>C	p.(Leu212Pro)	het	—	Likely pathogenic	PM1+PM2+PP1+PP3	Deleterious	Probably damaging	High	De novo	Yes
D	c.183delG	p.(Ala62Argfs∗22)	het	—	Pathogenic	PVS1+PM2+PP1				De novo	Yes

^∗^Allele frequency in East Asia reported by ExAC. hom: homozygous; het: heterozygous; —: no data. Notes: PVS1: null variant (nonsense, frameshift, canonical ±1 or 2 splice sites, initiation codon, single, or multiexon deletion) in a gene where loss of function (LOF) is a known mechanism of disease; PM1: located in a mutational hot spot and/or critical and well-established functional domain (e.g., active site of an enzyme) without benign variation; PM2: a variant is absent from a large general population or a control cohort; PM5: novel missense change at an amino acid residue where a different missense change determined to be pathogenic has been seen before; PP1: segregation of a variant in a family; PP3: multiple lines of computational evidence support a deleterious effect on the gene or gene product.

**Table 2:** Summary of all reported pathogenic variants in *POU4F3.*

Number	Nucleotide change	Protein change	Exon	Domain	Onset age of hearing loss	Progression	Prevalence	Origin	Audiometric configuration	Reference
1	Whole deletion of *POU4F3*				11~13 yo	Yes	N/A	Brazil	Flat and HF	Freitas et al. [13]
2	c.74dupA	p.His25fs∗18	1		~20 yo	Yes	15/602	Japan	HF	Kitano et al. [14]
3	c.120+1G>C		1		0~40 yo	Yes	3/16	China	Flat	He et al. [15]
4	c.183delG	p.A62Rfs∗22	2		25~44 yo	Yes	N/A	China	HF	This study
5	c.191A>T	p.Asp64Val	2		~30 yo	Yes	15/602	Japan	HF	Kitano et al. [14]
6	c.325C>T	p.His109Tyr	2		7~30 yo	Yes	N/A	China	HF	This study
7	c.337C>T	p.Gln113Ter	2		14~40 yo	Yes	N/A	China		Zhang et al. [16]
8	c.367delA	p.Ile123fs∗3	2		~40 yo	Yes	15/602	Japan	MF	Kitano et al. [14]
9	c.427C>T	p.Gln143Ter	2		3 yo	N/A	15/602	Japan	MF	Kitano et al. [14]
10	c.491C>G	p.Pro164Arg	2		N/A	N/A	1/6	China	Flat and HF	Wei et al. [17]
11	c.574G>T	p.Glu192Ter	2	POU	17~30 yo	Yes	15/602	Japan	HF	Kitano et al. [14]
12	c.581T>A	p.Phe194Tyr	2	POU	20 yo	Yes	15/602	Japan	HF	Kitano et al. [14]
13	c.602T>C	p.Leu201Pro	2	POU	>10 yo	Yes	N/A	China	MF	Gao et al. [12]
14	c.602delT	p.Leu201fs∗3	2	POU	16~30 yo	Yes	N/A	China	HF	Cai et al. [18]
15	c.603_604delGG	p.Val203Aspfs∗11	2	POU	N/A	N/A	N/A	China	N/A	Yang et al. [19]
16	c.635T>C	p.Leu212Pro	2	POU	10~20 yo	Yes	N/A	China	MF	This study
17	c.662_675del14	p.Gly221Glufsf∗14	2	POU	20 yo	N/A	1/42	Korea	HF	Lee et al. [20]
18	c.665C>T	p.Ser222Leu	2	POU	6 yo	Yes	15/602	Japan	HF	Kitano et al. [14]
19	c.668T>C	p.Leu223Pro	2	POU	13~20 yo	Yes	N/A	Netherlands	Flat, MF, and HF	Collin et al. [21]
20	c.680delC	p.Thr227fs∗13	2	POU	0 yo	Yes	15/602	Japan	MF	Kitano et al. [14]
21	c.694G>A	p.Glu232Lys	2	POU	~20 yo	N/A	1/8	Korea	HF	Baek et al. [22]
22	c.696G>T	p.Glu232Asp	2	POU	7~22 yo	Yes	N/A	China	HF	This study
23	c.718A>T	p.Asn240Tyr	2	POU	6 yo	Yes	15/602	Japan	MF	Kitano et al. [14]
24	c.841A>G	p.Ile281Val	2	POU homeobox	50~54 yo	Yes	15/602	Japan	HF	Kitano et al. [14]
25	c.865C>T	p.Leu289Phe	2	POU homeobox	13~20 yo	Yes	N/A	Netherlands	Flat, MF, and HF	Collin et al. [21]
26	c.884_891del8	Ile295Thrfs∗5	2	POU homeobox	18~30 yo	Yes	N/A	Israel	HF	Vahava et al. [11]
27	c.896C>T	p.Pro299Leu	2	POU homeobox	26~41 yo	Yes	15/602	Japan	MF	Kitano et al. [14]
28	c.932T>C	p.Leu311Pro	2	POU homeobox	10~20 yo	Yes	3/16	China	HF	He et al. [2]
29	c.976A>T	p.Arg326Ter	2	POU homeobox	Childhood	Yes	15/602	Japan	HF	Kitano et al. [14]
30	c.977G>A	p.Arg326Lys	2	POU homeobox	10~50 yo	N/A	N/A	Korea	HF	Kim et al. [41]
31	c.982A>G	p.Lys328Glu	2	POU homeobox	N/A	Yes	N/A	Taiwan	HF	Lin et al. [23]
32	c.1007delC	p.Ala336fs∗	2	POU homeobox	0 yo	Yes	1/3	Japan	N/A	Mutai et al. [24]

yo: years old; HF: high frequency; MF: middle frequency; N/A: not available.

## Data Availability

The patient's phenotype and the detected variants have been submitted to ClinVar (https://www.ncbi.nlm.nih.gov/clinvar/), and the Submission ID is SUB7170390.

## Supplementary Materials

Supplementary Materials In this study, we identified four novel variants using NGS of a panel of 168 deafness genes from four different Chinese families suffering from ADNSHL. The 168 deafness genes of the panel are listed concretely in Supplementary Table 1.

## References

[1] Liu L., Chen Y., Qi J. (2016). Wnt activation protects against neomycin-induced hair cell damage in the mouse cochlea. *Cell Death & Disease*.

[2] He Z., Guo L., Shu Y. (2017). Autophagy protects auditory hair cells against neomycin-induced damage. *Autophagy*.

[3] Zhu C., Cheng C., Wang Y. (2018). Loss of ARHGEF6 Causes Hair Cell Stereocilia Deficits and Hearing Loss in Mice. *Frontiers in Molecular Neuroscience*.

[4] Liu W., Xu X., Fan Z. (2019). Wnt Signaling Activates TP53-Induced Glycolysis and Apoptosis Regulator and Protects Against Cisplatin-Induced Spiral Ganglion Neuron Damage in the Mouse Cochlea. *Antioxidants & Redox Signaling*.

[5] He Z. H., Zou S. Y., Li M. (2020). The nuclear transcription factor FoxG1 affects the sensitivity of mimetic aging hair cells to inflammation by regulating autophagy pathways. *Redox Biology*.

[6] Smith R. J. H., Bale J. F., White K. R. (2005). Sensorineural hearing loss in children. *Lancet*.

[7] Kremer H. (2019). Hereditary hearing loss; about the known and the unknown. *Hearing Research*.

[8] Shearer A. E., Hildebrand M. S., Smith R. J. H. (2017). Hereditary hearing loss and deafness overview. *GeneReviews®[Internet]*.

[9] Cox B. C., Chai R., Lenoir A. (2014). Spontaneous hair cell regeneration in the neonatal mouse cochlea in vivo. *Development*.

[10] Weiss S., Gottfried I., Mayrose I. (2003). The DFNA15 deafness mutation affects POU4F3 protein stability, localization, and transcriptional activity. *Molecular and Cellular Biology*.

[11] Vahava O., Morell R., Lynch E. D. (1998). Mutation in transcription factor POU4F3 associated with inherited progressive hearing loss in humans. *Science*.

[12] Gao X., Xu J. C., Wang W. Q. (2018). A Missense Mutation in POU4F3 Causes Midfrequency Hearing Loss in a Chinese ADNSHL Family. *BioMed Research International*.

[13] Freitas É. L., Oiticica J., Silva A. G., Bittar R. S. M., Rosenberg C., Mingroni-Netto R. C. (2014). Deletion of the entire *POU4F3* gene in a familial case of autosomal dominant non-syndromic hearing loss. *European Journal of Medical Genetics*.

[14] Kitano T., Miyagawa M., Nishio S.-y. (2017). POU4F3 mutation screening in Japanese hearing loss patients: Massively parallel DNA sequencing-based analysis identified novel variants associated with autosomal dominant hearing loss. *PLOS ONE*.

[15] He L., Pang X., Chen P., Wu H., Yang T. (2016). Mutation in the hair cell specific gene POU4F3 is a common cause for autosomal dominant nonsyndromic hearing loss in Chinese Hans. *Neural Plasticity*.

[16] Zhang C., Wang M., Xiao Y. (2016). A novel nonsense mutation of POU4F3 gene causes autosomal dominant hearing loss. *Neural Plasticity*.

[17] Wei Q., Zhu H., Qian X. (2014). Targeted genomic capture and massively parallel sequencing to identify novel variants causing Chinese hereditary hearing loss. *Journal of Translational Medicine*.

[18] Cai X. Z., Li Y., Xia L. (2017). Exome sequencing identifies *POU4F3* as the causative gene for a large Chinese family with non-syndromic hearing loss. *Journal of Human Genetics*.

[19] Yang T., Wei X., Chai Y., Li L., Wu H. (2013). Genetic etiology study of the non-syndromic deafness in Chinese Hans by targeted next-generation sequencing. *Orphanet Journal of Rare Diseases*.

[20] Lee H. K., Park H. J., Lee K. Y., Park R., Kim U. K. (2010). A novel frameshift mutation of *POU4F3* gene associated with autosomal dominant non-syndromic hearing loss. *Biochemical and Biophysical Research Communications*.

[21] Collin R. W. J., Chellappa R., Pauw R. J. (2008). Missense mutations in POU4F3 cause autosomal dominant hearing impairment DFNA15 and affect subcellular localization and DNA binding. *Human Mutation*.

[22] Baek J. I., Oh S. K., Kim D. B. (2012). Targeted massive parallel sequencing: the effective detection of novel causative mutations associated with hearing loss in small families. *Orphanet Journal of Rare Diseases*.

[23] Lin Y. H., Lin Y. H., Lu Y. C. (2017). A novel missense variant in the nuclear localization signal of *POU4F3* causes autosomal dominant non-syndromic hearing loss. *Scientific Reports*.

[24] Mutai H., Suzuki N., Shimizu A. (2013). Diverse spectrum of rare deafness genes underlies early-childhood hearing loss in Japanese patients: a cross-sectional, multi-center next-generation sequencing study. *Orphanet Journal of Rare Diseases*.

[25] Gao X., Yuan Y. Y., Lin Q. F. (2018). Mutation ofIFNLR1, an interferon lambda receptor 1, is associated with autosomal-dominant non-syndromic hearing loss. *Journal of Medical Genetics*.

[26] Richards S., on behalf of the ACMG Laboratory Quality Assurance Committee, Aziz N. (2015). Standards and guidelines for the interpretation of sequence variants: a joint consensus recommendation of the American College of Medical Genetics and Genomics and the Association for Molecular Pathology. *Genetics in Medicine*.

[27] Oza A. M., DiStefano M. T., Hemphill S. E. (2018). Expert specification of the ACMG/AMP variant interpretation guidelines for genetic hearing loss. *Human Mutation*.

[28] Wang T., Chai R., Kim G. S. (2015). Lgr5+ cells regenerate hair cells via proliferation and direct transdifferentiation in damaged neonatal mouse utricle. *Nature Communications*.

[29] Zhang S., Zhang Y., Dong Y. (2020). Knockdown of Foxg1 in supporting cells increases the trans-differentiation of supporting cells into hair cells in the neonatal mouse cochlea. *Cellular and Molecular Life Sciences*.

[30] Lu X., Sun S., Qi J. (2017). Bmi 1 regulates the proliferation of cochlear supporting cells via the canonical Wnt signaling pathway. *Molecular Neurobiology*.

[31] Tan F., Chu C., Qi J. (2019). AAV-ie enables safe and efficient gene transfer to inner ear cells. *Nature Communications*.

[32] Cheng C., Wang Y., Guo L. (2019). Age-related transcriptome changes in Sox2+ supporting cells in the mouse cochlea. *Stem Cell Research & Therapy*.

[33] Zhang S., Liu D., Dong Y. (2019). Frizzled-9+ supporting cells are progenitors for the generation of hair cells in the postnatal mouse cochlea. *Frontiers in Molecular Neuroscience*.

[34] Yan W., Liu W., Qi J. (2018). A three-dimensional culture system with Matrigel promotes purified spiral ganglion neuron survival and function in vitro. *Molecular Neurobiology*.

[35] ACMG Working Group on Update of Genetics Evaluation Guidelines for the Etiologic Diagnosis of Congenital Hearing Loss (2014). American College of Medical Genetics and Genomics guideline for the clinical evaluation and etiologic diagnosis of hearing loss. *Genetics in Medicine*.

[36] Yuan Y., You Y., Huang D. (2009). Comprehensive molecular etiology analysis of nonsyndromic hearing impairment from typical areas in China. *Journal of Translational Medicine*.

[37] Lin X., Tang W., Ahmad S. (2012). Applications of targeted gene capture and next-generation sequencing technologies in studies of human deafness and other genetic disabilities. *Hearing Research*.

[38] Idan N., Brownstein Z., Shivatzki S., Avraham K. B. (2013). Advances in genetic diagnostics for hereditary hearing loss. *Journal of Basic and Clinical Physiology and Pharmacology*.

[39] Sloan-Heggen C. M., Bierer A. O., Shearer A. E. (2016). Comprehensive genetic testing in the clinical evaluation of 1119 patients with hearing loss. *Human Genetics*.

[40] Yuan Y., Li Q., Su Y. (2020). Comprehensive genetic testing of Chinese SNHL patients and variants interpretation using ACMG guidelines and ethnically matched normal controls. *European Journal of Human Genetics*.

[41] Kim H. J., Won H. H., Park K. J. (2013). SNP linkage analysis and whole exome sequencing identify a novel POU4F3 mutation in autosomal dominant late-onset nonsyndromic hearing loss (DFNA15). *PLoS One*.

[42] Wegner M., Drolet D. W., Rosenfeld M. G. (1993). POU-domain proteins: structure and function of developmental regulators. *Current Opinion in Cell Biology*.

[43] Herr W., Cleary M. A. (1995). The POU domain: versatility in transcriptional regulation by a flexible two-in-one DNA-binding domain. *Genes & Development*.

[44] Xiang M., Gan L., Li D. (1997). Essential role of POU-domain factor Brn-3c in auditory and vestibular hair cell development. *Proceedings of the National Academy of Sciences of the United States of America*.

[45] Keithley E. M., Erkman L., Bennett T., Lou L., Ryan A. F. (1999). Effects of a hair cell transcription factor, Brn-3.1, gene deletion on homozygous and heterozygous mouse cochleas in adulthood and aging. *Hearing Research*.

[46] Hertzano R., Montcouquiol M., Rashi-Elkeles S. (2004). Transcription profiling of inner ears from Pou4f3ddl/ddl identifies Gfi1 as a target of the Pou4f3 deafness gene. *Human Molecular Genetics*.

[47] Klauer A. A., van Hoof A. (2012). Degradation of mRNAs that lack a stop codon: a decade of nonstop progress. *Wiley Interdisciplinary Reviews: RNA*.

[48] Costa A., Powell L. M., Lowell S., Jarman A. P. (2017). Atoh1 in sensory hair cell development: constraints and cofactors. *Seminars in Cell & Developmental Biology*.

[49] Wallis D., Hamblen M., Zhou Y. (2003). The zinc finger transcription factor Gfi1, implicated in lymphomagenesis, is required for inner ear hair cell differentiation and survival. *Development*.

[50] Chonko K. T., Jahan I., Stone J. (2013). *Atoh1* directs hair cell differentiation and survival in the late embryonic mouse inner ear. *Developmental Biology*.

[51] Ma D. B., Chen J., Xia Y. (2014). Inhibition of Myo6 gene expression by co-expression of a mutant of transcription factor POU4F3 (BRN-3C) in hair cells. *Molecular Medicine Reports*.

[52] Tornari C., Towers E. R., Gale J. E., Dawson S. J. (2014). Regulation of the orphan nuclear receptor Nr2f2 by the DFNA15 deafness gene Pou4f3. *PLoS One*.

