# Supplementary material for: Four Novel Variants in POU4F3 Cause Autosomal Dominant Nonsyndromic Hearing Loss
Source: Neural Plast. 2020 Jul 1;2020:6137083. doi: 10.1155/2020/6137083 (PMC7349627; doi:10.1155/2020/6137083)
Supplement: Supplementary Materials — In this study, we identified four novel variants using NGS of a panel of 168 deafness genes from four different Chinese families suffering from ADNSHL. The 168 deafness genes of the panel are listed concretely in Supplementary Table 1. [file 6137083.f1.docx]

**Supplementary Table 1**

List of the 168 deafness genes and miRNA

| ACTG1 | ALX3 | BSND | CABP2 | CCDC50 | CDH23 | CEACAM16 |
| --- | --- | --- | --- | --- | --- | --- |
| CHD7 | CIB2 | CLDN14 | CLPP | CLRN1 | COCH | COL11A1 |
| COL11A2 | COL1A1 | COL1A2 | COL2A1 | COL4A3 | COL4A4 | COL4A5 |
| COL4A6 | COL9A1 | COL9A2 | CRYM | DFNA5 | DFNB31 | DFNB59 |
| DIABLO | DIAPH1 | DIAPH3 | DSPP | ECM1 | EDN3 | EDNRB |
| ELMOD3 | ESPN | ESRRB | EYA1 | EYA4 | FGF3 | FGF8 |
| FGFR1 | FGFR3 | FLNA | FOXI1 | FREM1 | FXN | GATA3 |
| GIPC3 | GJB1 | GJB2 | GJB3 | GJB6 | GLYAT | GPR98 |
| GPSM2 | GRHL2 | GRXCR1 | HARS | HARS2 | HGF | HMX1 |
| HOXA2 | HSD17B4 | IL13 | ILDR1 | KARS | KCNE1 | KCNJ10 |
| KCNQ1 | KCNQ4 | KITLG | KRT9 | LAMA3 | LARS2 | LHFPL5 |
| LOXHD1 | LRTOMT | MARVELD2 | MIR96 | MITF | MPZ | MSRB3 |
| MYH14 | MYH9 | MYO15A | MYO1A | MYO1E | MYO3A | MYO6 |
| MYO7A | NDP | NDRG1 | NEFL | NELL2 | NF2 | OPA1 |
| OTOA | OTOF | OTOG | OTOGL | P2RX2 | PABPN1 | PAX3 |
| PCDH15 | PCDH9 | PDZD7 | PMP22 | PNPT1 | POLR1C | POLR1D |
| POU3F4 | POU4F3 | PROK2 | PROKR2 | PRPS1 | PTPN11 | PTPRQ |
| PTPRR | RDX | RPGR | SALL1 | SALL4 | SEC23A | SEMA3E |
| SERPINB6 | SIX1 | SIX5 | SLC17A8 | SLC19A2 | SLC26A4 | SLC26A5 |
| SMAD4 | SMPX | SNAI2 | SOX10 | STRC | TBC1D24 | TCIRG1 |
| TCOF1 | TECTA | TIMM8A | TJP2 | TMC1 | TMEM126A | TMIE |
| TMPRSS3 | TMPRSS4 | TNC | TPRN | TRIOBP | TRMU | TSPEAR |
| TYR | USH1C | USH1G | USH2A | WFS1 |  |  |

| MT-RNR1 | chrM:640-1601 |
| --- | --- |
| MT-TL1 | chrM:3230-3304 |
| MT-CO1 | chrM:5904-7445 |
| MT-TS1 | chrM:7446-7514 |
| MT-TK | chrM:8295-8364 |
| MT-TE | chrM:14674-14742 |

| miR-96 | chr7: 129414532-129414609 |
| --- | --- |
| miR-182 | chr7: 129410223-129410332 |
| miR-183 | chr7: 129414745-129414854 |
